# Dry pleurisy complicating solitary pulmonary nodules caused by *Mycobacterium avium*: a case report

**DOI:** 10.1186/s13256-015-0723-4

**Published:** 2015-10-26

**Authors:** Takanori Asakura, Makoto Ishii, Mizuha Haraguchi, Ikuo Kamiyama, Mitsutomo Kohno, Hiroyuki Sakamaki, Katsura Emoto, Yuichiro Hayashi, Hiroaki Sugiura, Ichiro Kawada, Kenzo Soejima, Ho Namkoong, Sadatomo Tasaka, Naoki Hasegawa, Tomoko Betsuyaku

**Affiliations:** 1 Division of Pulmonary Medicine, Department of Medicine, Keio University School of Medicine, 35 Shinanomachi, Shinjuku, Tokyo, 160-8582 Japan; 2 Department of Medicine, Nippon Kokan Hospital, 1-2-1 Kokan-dori, Kawasaki, Kanagawa 210-0852 Japan; 3 Division of Thoracic Surgery, Department of Surgery, Keio University School of Medicine, 35 Shinanomachi, Shinjuku, Tokyo, 160-8582 Japan; 4 Division of Diagnostic Pathology, Keio University Hospital, 35 Shinanomachi, Shinjuku, Tokyo, 160-8582 Japan; 5 Department of Diagnostic Radiology, Keio University School of Medicine, 35 Shinanomachi, Tokyo, Shinjuku 160-8582 Japan; 6 Center for Infectious Diseases and Infection Control, Keio University School of Medicine, 35 Shinanomachi, Shinjuku, Tokyo, 160-8582 Japan

**Keywords:** *Mycobacterium avium* complex lung disease, solitary pulmonary nodule, ^18^F-fluorodeoxyglucose, positron emission tomography

## Abstract

**Introduction:**

*Mycobacterium avium* complex (MAC) lung disease presenting as a solitary pulmonary nodule (MAC-SPN) is often asymptomatic, is more common in middle to old age, and mimics lung cancer or tuberculoma. We report herein a case of MAC-SPN in an immunocompetent young adult patient, presenting with persistent chest pain and a subacutely progressive nodule with high intense ^18^F-fluorodeoxyglucose uptake. Histological examination of resected specimens revealed pleurisy, which is a rare finding of MAC-SPN.

**Case presentation:**

A 36-year-old Japanese male presented with chest pain and a subacutely progressive pulmonary nodule. Positron emission tomography-computed tomography showed high intense ^18^F-fluorodeoxyglucose uptake in the nodule. Owing to his continuous chest pain and subacutely progressive nodules, wedge resection was performed using video-assisted thoracoscopic surgery. Histological examination revealed an epithelioid granuloma and pleurisy, and the lung tissue culture was positive for mycobacteria identified as *M. avium*.

**Conclusion:**

This is the first report of MAC-SPN occurring with persistent chest pain, suggesting that MAC should be considered in the differential diagnosis of a solitary pulmonary nodule, even for patients who experience persistent chest pain. As in the present case, surgical resection with video-assisted thoracoscopic surgery is a reasonable approach to the diagnosis and treatment of MAC-SPN with possible malignancy, especially as MAC can be diagnosed using resected lung tissue culture with histological confirmation.

## Introduction

*Mycobacterium avium* complex (MAC) lung disease commonly presents as upper lobe fibrocavitary or nodular bronchiectatic forms of pulmonary disease and occasionally presents as a solitary pulmonary nodule (MAC-SPN) [1]. MAC-SPN is often asymptomatic, is more common in middle to old age, and mimics lung cancer or tuberculoma [2–4]. We report a case of MAC-SPN in an immunocompetent young adult patient, presenting with persistent chest pain and a subacutely progressive nodule with high intense ^18^F-fluorodeoxyglucose (FDG) uptake. Histological examination of resected specimens revealed pleurisy, which is a rare finding of MAC-SPN.

## Case presentation

**Fig. 1:**
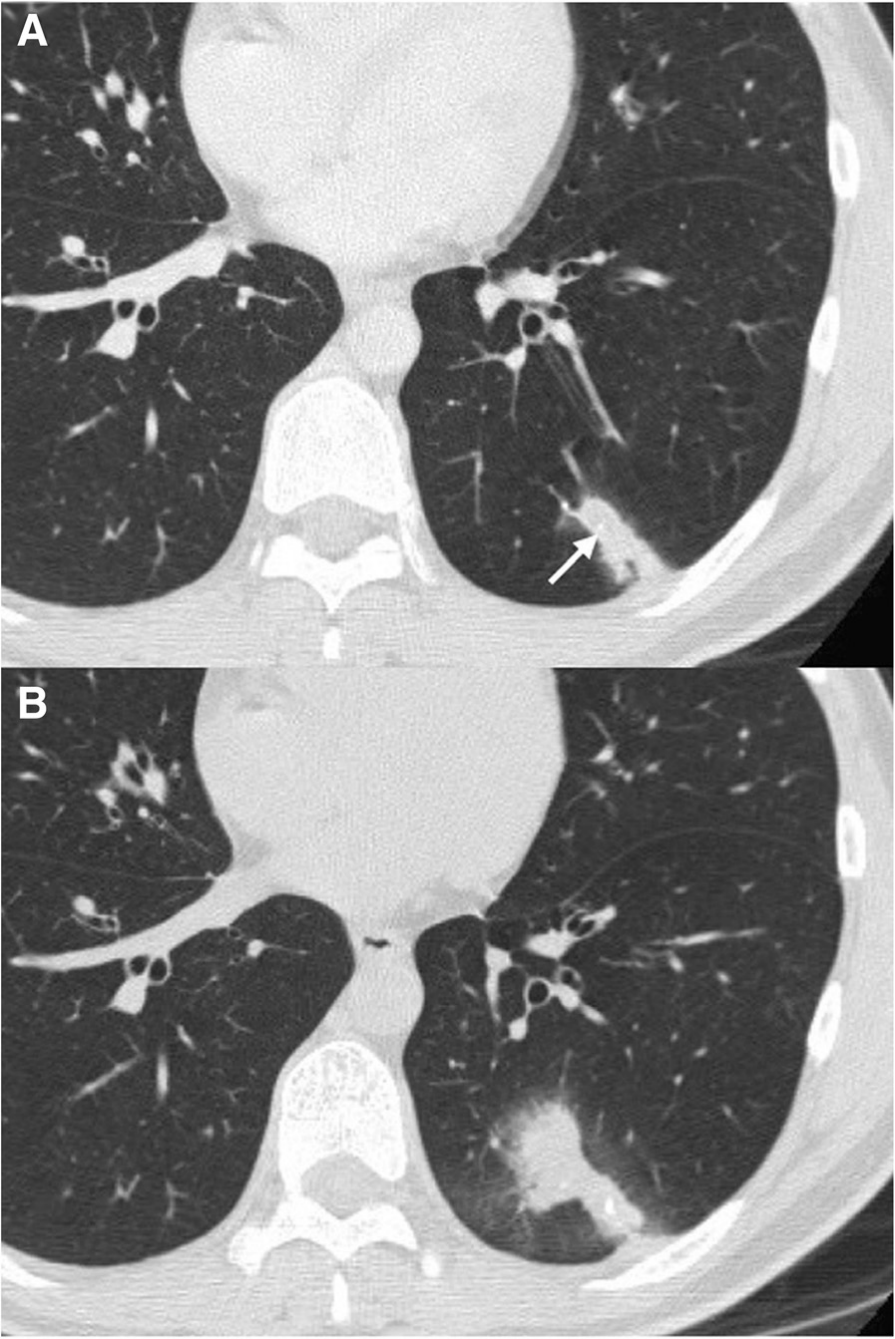
Computed tomography scan of the chest. **a** Chest computed tomography showed an irregularly shaped solitary nodule in the periphery of the left lower lobe, with microcalcifications (arrow) and pleural indentation. **b** After 2 weeks, the nodules became enlarged, around which ground glass opacity appeared

A 36-year-old Japanese male was referred to our hospital because of persistent chest pain and a subacutely progressive pulmonary nodule. Four weeks prior to his referral to our hospital, he began to experience chest pain that increased in severity on inspiration. He visited a private hospital the following day. He had no family or personal history of nontuberculous or pulmonary disease. Chest computed tomography (CT) showed an irregularly shaped solitary nodule in the periphery of the left lower lobe with microcalcifications and pleural indentation. On CT at a follow-up appointment 2 weeks after the initial consultation, the nodule was enlarged (Fig. 1a, b). To diagnose the subacutely progressive pulmonary nodule, a CT-guided lung biopsy was performed in the private hospital. The histological examination of the biopsy specimens revealed granulomatous inflammation with atypical cells of various sizes. One week later, the patient was referred to our hospital. Although we intended to clarify the origin of the atypical cells and rule out malignancy, the specimen volume was insufficient for further investigation.

**Fig. 2:**
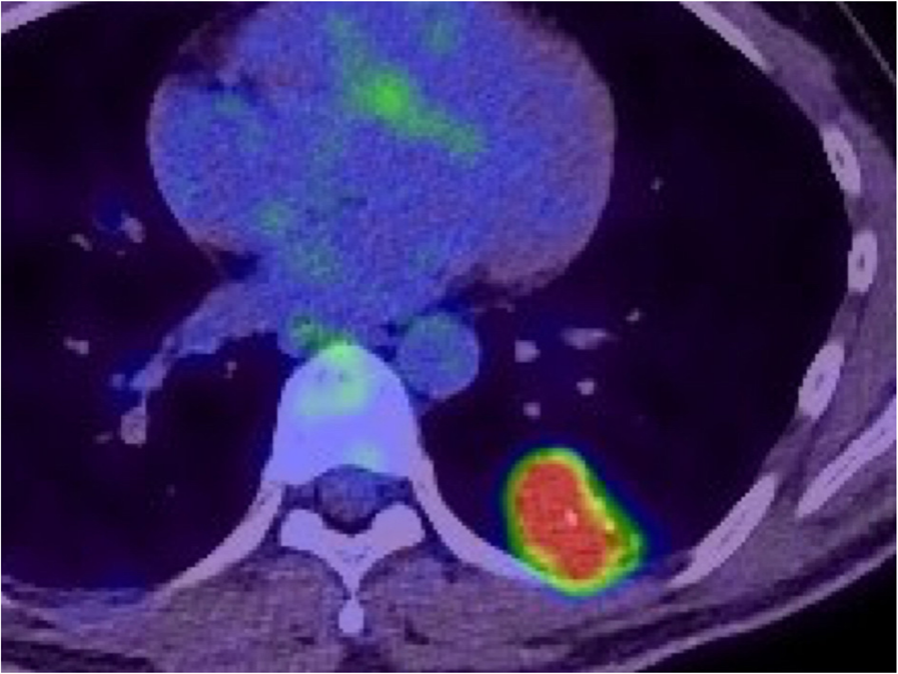
Positron emission tomography-computed tomography scan of the chest. A positron emission tomography-computed tomography scan showed intense ^18^F-fluorodeoxyglucose uptake in the nodular shadow with a maximum standard uptake value of 13.9

He reported no history of smoking, foreign travel, eating crab or boar or deer meat, or contact with patients with active pulmonary tuberculosis. The results of his physical examinations, including vital signs and consciousness, were normal. The laboratory data, including lymphocyte count, C-reactive protein, procalcitonin, gamma globulin, tumor markers, antineutrophil cytoplasmic antibodies, anti-HIV antibody, β-D-glucan, aspergillus antigen, cryptococcus antigen, Interferon-Gamma Release Assay (T-SPOT.TB®), and antibody to *Paragonimus westermani* or *P. miyazaki*, were unremarkable. Two weeks after the referral, positron emission tomography (PET)-CT showed intense FDG uptake in the nodules, with a maximum standard uptake value (SUV) of 13.94 (Fig. 2).

**Fig. 3:**
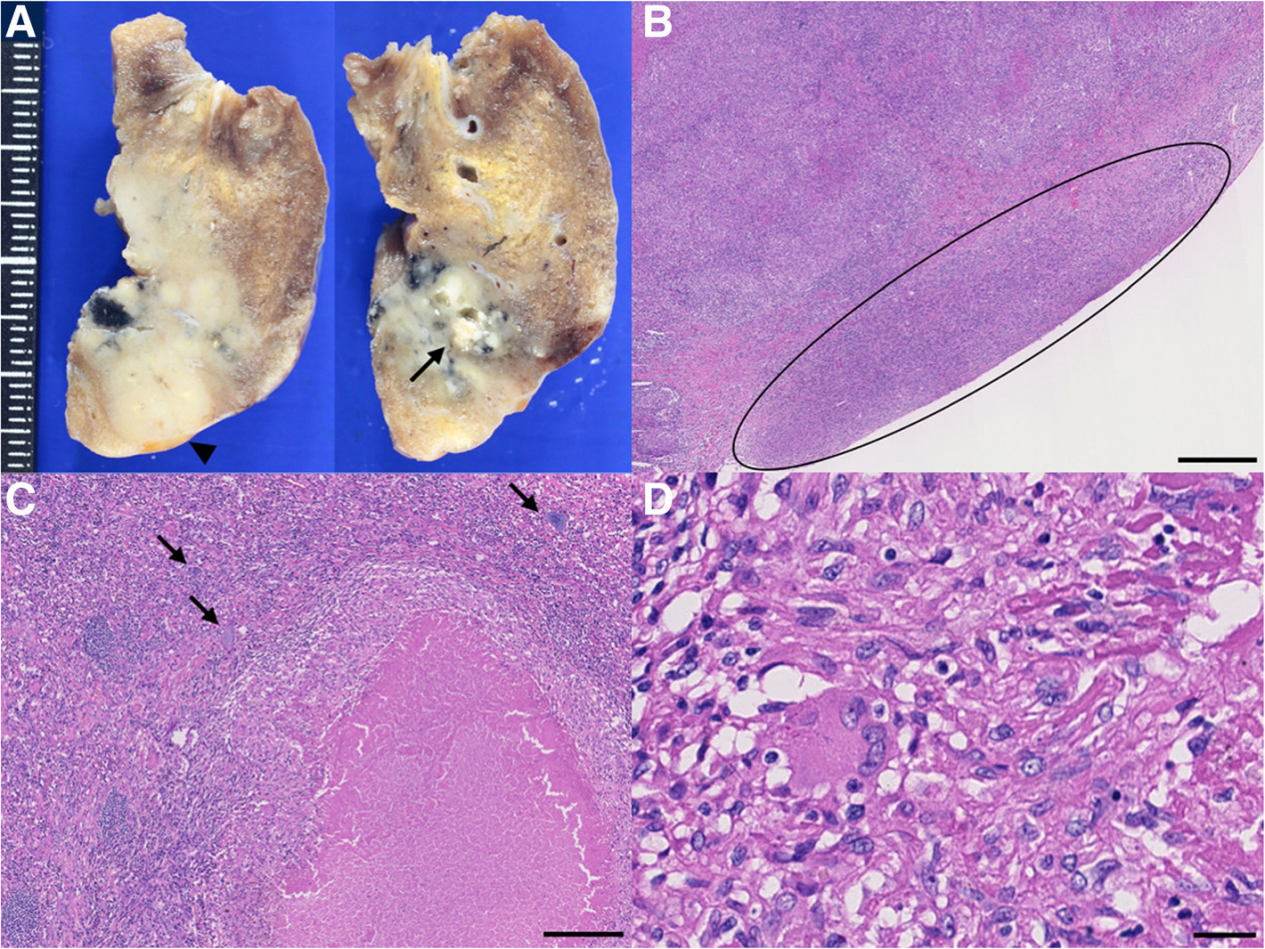
Photograph and photomicrographs of the lung. **a** Photograph of a cross-sectional specimen in the resected lung showing a granuloma with caseating necrosis (arrow) and pleurisy (arrowhead). **b** Photomicrograph showing pleurisy over the visceral pleurae (circle; bar 500 μm), which revealed granulomatous infiltration. **c**-**d** Photomicrograph showing an epithelioid granuloma with necrosis and calcification (arrows) (C; bar 250 μm, D; bar 25 μm)

Owing to his continuous chest pain and subacutely progressive nodules, which could not be diagnosed using CT-guided lung biopsy, wedge resection via video-assisted thoracoscopic surgery was performed in the left lower lobe. The histological examination revealed an epithelioid granuloma, with caseating necrosis and calcification, and pleurisy over the visceral pleurae (Fig. 3a–d). Although a smear test of the resected lung tissue was negative, the patient was initially diagnosed with tuberculosis based on the results of the histological examination. Antituberculous therapy was initiated with isoniazid, rifampicin, ethambutol, and pyrazinamide. Because the patient presented with severe skin eruptions and high fever 2 weeks after initiation of the antituberculous therapy, we paused the treatment. One week later, the lung tissue culture was positive for mycobacteria identified as *Mycobacterium avium* via DNA-DNA hybridization. His chest pain disappeared almost simultaneously. Anti-interferon-γ autoantibodies were negative. We plan to initiate anti-MAC chemotherapy (rifampicin, ethambutol, and clarithromycin) once these adverse effects have abated, for which the patient is being closely monitored.

## Discussion

To our knowledge, this is the first report regarding MAC-SPN presenting with persistent chest pain and induced by histologically confirmed pleurisy. Since Gribetz *et al*. reported the first case series in 1981 [1], 45 cases of MAC-SPN have been reported: 33 from East Asia [2–10] and 12 from the USA [1]. MAC-SPN generally affects middle-aged to elderly (median = 63 years; range: 37–81 years) men and women equally. The majority of the cases were diagnosed via chest imaging, and the patients were usually asymptomatic; however, respiratory symptoms, such as cough, sputum, and hemoptysis, were reported occasionally. In contrast, our patient was younger and had no respiratory symptoms aside from persistent chest pain, which was induced by histologically confirmed pleurisy without pleural effusion (so-called “dry pleurisy”) caused by *M. avium*. A report of MAC (not MAC-SPN) pleurisy in 16 cases with pleural effusion included a patient who was younger than the patient in the present case [11]. The factors for the onset of MAC-SPN are unknown. In the literature, only 7 cases were associated with underlying pulmonary disease (4 with old tuberculosis, 2 with lung cancer, and 1 with emphysema), and none of the patients were immunocompromised or had a family history of MAC lung disease. However, given that recent studies have suggested the importance of genetic or familial susceptibility to MAC lung disease [12, 13], further studies are warranted to assess MAC-SPN host factors.

In the present case, lung cancer and tuberculoma were included in the differential diagnosis of SPN prior to the definitive diagnosis of MAC-SPN. Distinguishing MAC from tuberculoma in cases involving an SPN is very difficult. In a report of 26 patients with tuberculoma and 15 patients with MAC-SPN [8], 10 of the patients with MAC-SPN were initially diagnosed with tuberculoma and administered antituberculous medications. The patients with MAC-SPN were older and had higher maximum SUV in PET than the patients with tuberculoma. In 9 cases of nontuberculous mycobacterium (NTM) presenting as an SPN [7–9], the mean maximum SUV on FDG-PET was 8.6 (range: 4.4–13.2). Therefore, the maximum SUV in the present case (13.9) was higher than that observed in previous reports. Ruling out lung cancer should be undertaken with great care, as the coexistence of lung cancer and MAC pulmonary disease has been reported [2, 14]. FDG-PET is reportedly useful for evaluating disease activity and monitoring the therapeutic response in patients with NTM lung disease [15], indicating that high activity of MAC-SPN could cause pleurisy, as observed in the present case.

In the present case, surgical resection of MAC-SPN was performed, and we plan to initiate anti-MAC chemotherapy once the adverse effects have abated, although the significance of chemotherapy for MAC-SPN following surgical resection remains controversial. In the literature, of 45 patients with MAC lung disease presenting as an SPN, surgical resection was performed in 16 patients. Of the 14 patients who were administered anti-MAC chemotherapy after the surgical resection, none experienced recurrence; however, this was also true for the remaining 2 patients who were not administered anti-MAC chemotherapy [1–5, 7]. Chemotherapy for MAC-SPN without surgical resection has also been reported as effective, with the size of the SPN decreasing in 9 of 10 patients undergoing anti-MAC chemotherapy [10]. In 15 patients with MAC-SPN, 5 did not undergo any treatment, and the size of the SPN decreased spontaneously in 3 patients, was unchanged in 1 patient, and increased in 1 patient. Anti-MAC chemotherapy was eventually initiated in 2 of these 5 patients [8]. Therefore, anti-MAC chemotherapy can be withheld until the size of an SPN increases, even without surgical therapy.

## Conclusion

We report a case with MAC-SPN with high-intensity FDG uptake and histologically confirmed pleurisy. MAC should be considered in the differential diagnosis of an SPN, even for patients who experience persistent chest pain. Surgical resection with video-assisted thoracoscopic surgery is a reasonable approach to the diagnosis and treatment of MAC-SPN with possible malignancy, as MAC can be diagnosed using resected lung tissue culture with histological confirmation.

## Consent

Written informed consent was obtained from the patient for publication of this case report and accompanying images. A copy of the written consent is available for review by the Editor-in-Chief of this journal.

## Abbreviations

CT: computed tomography
FDG: ^18^F-fluorodeoxyglucose
MAC: *Mycobacterium avium* complex
NTM: nontuberculous mycobacterium
PET: positron emission tomography
SUV: standard uptake value
SPN: solitary pulmonary nodule
